# Variable and slow-paced neural dynamics in HVC underlie plastic song production in juvenile zebra finches

**DOI:** 10.1186/s12868-024-00915-7

**Published:** 2024-12-23

**Authors:** Linda Bistere, Stefan Wilczek, Daniela Vallentin

**Affiliations:** 1 Max Planck Institute for Biological Intelligence, Eberhard-Gwinner-Str., 82319 Seewiesen, Germany; 2 Graduate School for Systemic Neurosciences GSN-LMU, Munich, Germany

**Keywords:** Vocal learning, HVC, Songbirds

## Abstract

Zebra finches undergo a gradual refinement of their vocalizations, transitioning from variable juvenile songs to the stereotyped song of adulthood. To investigate the neural mechanisms underlying song crystallization—a critical phase in this developmental process—we performed intracellular recordings in HVC (a premotor nucleus essential for song learning and production) of juvenile birds. We then compared these recordings to previously published electrophysiological data from adult birds. We found that HVC projection neurons in juvenile zebra finches during the song crystallization phase exhibited more variable spiking patterns compared to the precise bursting observed in adult HVC projection neurons. Additionally, subthreshold membrane potential fluctuations in juvenile neurons exhibited longer durations and larger amplitude excitatory postsynaptic potentials. These distinct temporal dynamics in HVC during song crystallization likely play a crucial role in the fine-tuning processes that shape the precise timing and structure of the mature zebra finch song.

## Introduction

The process of acquiring a motor skill involves refining a once highly variable movement into its finely-tuned and precise execution. For example, the initial stages of vocal learning in humans are often characterized by variable vocalizations such as cries and babbling sounds [1]. After months of practice, these utterances shape into refined words with precise pronunciation [2]. Similarly, male zebra finches undergo a comparable learning process for singing [3]. During a critical period, their initially variable subsong transitions into a plastic song, ultimately culminating in the acquisition of a final, stereotyped song that closely resembles that of their tutor [4].

Zebra finch song development involves a gradual refinement of vocalizations, transitioning from variable juvenile songs to a stable, stereotyped adult form. The song learning progression in developing zebra finches can be monitored by analyzing changes in both spectral (Wiener entropy, spectral continuity, pitch and frequency modulation) and temporal (duration of syllables and gaps) features of their vocalizations [4–6]. Subsong [~ 25–50 days post hatch (dph)] consists of poorly structured sounds with highly variable spectral features [7]. This phase is followed by the plastic song phase (~ 50–80 dph), which consists of structured syllable production that gradually develops distinct acoustic features until a stereotyped, crystalized song is achieved, with precise, reliably repeated features [7]. In adults, the spectral and temporal features of song are highly stereotyped, and acoustic parameters have low variability.

Throughout the developmental phase, neural dynamics in the vocal production pathway undergo various modifications [8–13], ultimately resulting in a stable neural pattern during song production [14–19]. Initially, the lateral magnocellular nucleus of the anterior neostriatum (LMAN) is necessary for the production and timing of subsong vocal patterns [8]. LMAN projects to RA (robust nucleus of the arcopallium), which in turn projects to the brainstem vocal and respiratory nuclei [20], leading to audible song production. During the song learning phase, the main input to RA switches from LMAN to HVC, which, on a behavioral level, is reflected by increased stereotypy in timing and spectral features of song [21]. This is a gradual and overlapping transition, as HVC inactivation during the plastic song phase reverts juveniles singing to subsong, whereas LMAN inactivation leads to the production of adult like stereotyped song sequences [10, 22].

We aimed to explore the neural underpinnings that might fine-tune temporal dynamics of plastic song, ultimately leading to a stereotyped song. Between 70 and 90 days post hatch, the temporal structure of the song changes; for instance, silent gap duration decreases, and the overall timing variability is reduced [23]. While RA is necessary for song production throughout all stages of development, its spiking pattern develops alongside spectral features [17], but it does not generate timing [24]. LMAN induces temporal variability, but only during the subsong phase, as after ~ 60 dph, the RA motor program is predominantly driven by HVC [8]. Although HVC is not necessary for subsong, it is essential for all later stages of song production and generates the timing of song in both adults [24] and juveniles. In juveniles, HVC lesions result in the abolition of adult like gap and syllable durations and HVC cooling in adults results in an elongation of syllables and gaps [13].

During the early plastic song phase, HVC projection neurons produce rhythmic bursts across several syllables [11]. As the song matures, the bursting activity becomes tied to a specific timepoint during song [11]; however, how these neural changes relate to alterations in the temporal features of singing behavior remains unclear. Here, we directly investigated neural activity changes at the single-cell level that underlie the transition from plastic song to crystallized song. We quantified the neural dynamics, including changes in spiking characteristics and the synaptic inputs generated by connecting neurons, by analyzing subthreshold membrane potential in singing zebra finches. Ultimately, the crystallized song is sung by adult males to attract females, who prefer a song with high temporal precision.

## Results

### HVC projection neurons in juveniles exhibit more variable spiking and bursting patterns compared to those in adults

To understand the neural mechanisms underlying this temporal refinement, we focused on HVC, a premotor nucleus crucial for song learning and production. In adult zebra finches, the HVC motor program for song consists of precise, time-locked bursts of activity [14–16], elicited by HVC projection neurons. These neurons either project to the robust nucleus of the arcopallium [14], playing a direct role in song production, or to the basal ganglia circuit—specifically area X—indirectly contributing to song production [25, 26]. We aimed to investigate how the motor program develops during the plastic song phase, a late stage in song learning.

To achieve this, we conducted intracellular recordings of seven HVC-RA neurons and three HVC-Area X neurons in four freely behaving male juvenile zebra finches (74–94 days post hatch) during singing. Given the HVC's role in controlling syllable and gap duration during this phase [13, 24], we analyzed the neural activity of HVC projection neurons in juveniles and compared our results with a previously recorded dataset from ten adult birds (26 HVC-RA neurons and 28 HVC-Area X neurons taken from Vallentin and Long [28]) to identify potential differences that might contribute to the ongoing refinement of song timing. As we did not observe significant differences between HVC-RA and HVC-Area X neuron activity, we pooled the data of all HVC projection neurons to increase statistical power. However, for data transparency, we clearly distinguish the different populations and provide separate data figures for HVC-RA neurons.

**Fig. 1:**
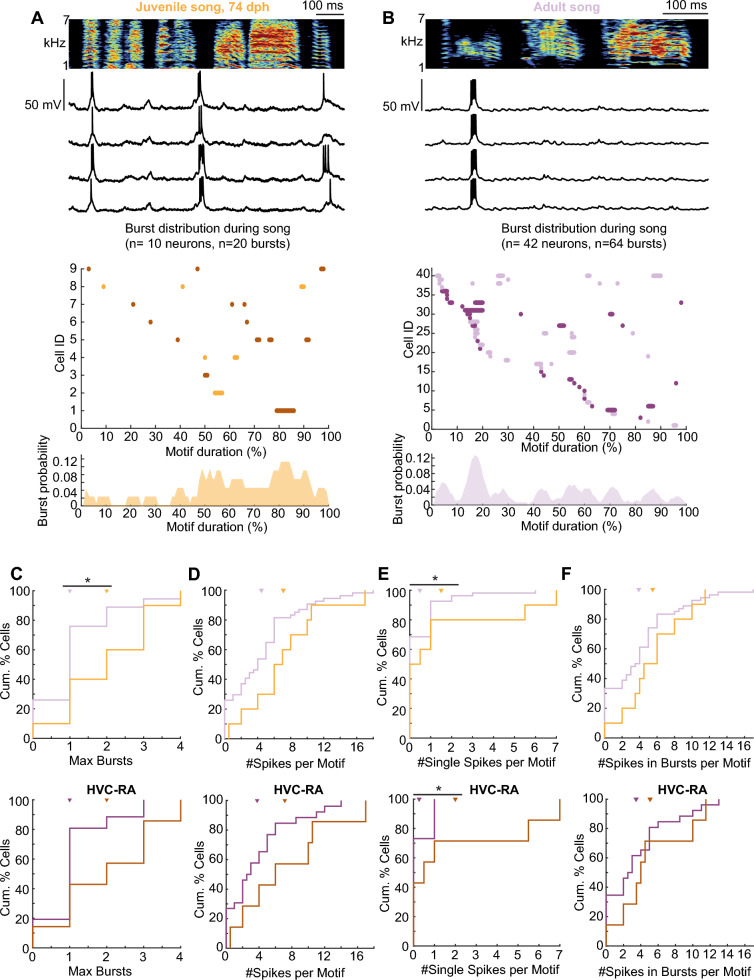
HVC projection neurons in juvenile birds burst frequently during song production. **A** Top: example recording of an HVC projection neuron (top: spectrogram of a song motif, below: intracellular recording of an HVC projection neuron during four repetitions of the song motif), Middle: Raster-plot of bursting activity during singing a motif (0–100% of duration) in all birds. Identified HVC-RA neurons are marked in dark shading. Bottom: probability distribution of burst occurrence during motif. **B** Same as in **A** but for adult birds. **C** (Top) maximum number of bursts of all HVC projection neurons during song production between juveniles (orange) and adults (purple). (Bottom) maximum number of bursts of HVC-RA neurons. Juveniles: 0–4 bursts per motif, 7 HVC-RA neurons, median = 2, MAD = 1.14, Adults: 0–3 bursts per motif, 26 HVC-RA neurons, median = 1, MAD = 0.57, Wilcoxon rank sum test, p = 0.01. **D** (Top) number of spikes of all HVC projection neurons per trial in juveniles (orange) and adults (purple). (Bottom) number of spikes of HVC-RA neurons. Juveniles mean ± standard deviation = 7.14 ± 5.74 spikes per motif, Adults mean ± standard deviation = 3.77 ± 3.92 spikes per motif, two-sample t-test, p = 0.08. **E** (Top) distribution of single spikes per trial across all recorded projection neurons in juveniles and adults. (Bottom) distribution of single spikes per trial across HVC-RA neurons. Juveniles mean ± standard deviation = 2 ± 2.96 single spikes per motif, Adults mean ± standard deviation = 0.27 ± 0.45, two-sample t-test, p = 0.005. **F** (Top) distribution of number of spikes of HVC projection neurons recruited for bursts per song motif. (Bottom) distribution of number of spikes of HVC-RA neurons. Juveniles mean ± standard deviation = 5.07 ± .18 spikes per burst, Adults mean ± standard deviation = 3.46 ± 3.73 spikes per burst, two-sample t-test, p = 0.33

We found that HVC projection neurons elicited sparse bursts of action potentials at several specific time points during song production (Fig. 1A, B). The distribution of bursts spanned the entire song motif in both age groups [15, 16] (Fig. 1A, B). This observation suggests that the neural dynamics underlying song production are established during the late phase of song learning, leading to the production of stereotyped song structure. However, this finding alone cannot account for the previously described differences in temporal variability between juvenile and adult songs [23], suggesting that additional factors contribute to the ongoing refinement of song timing.

In adult zebra finches, HVC projection neurons display a sparse number of bursts during song production [14–16, 24, 27, 28]. To explore whether our recorded neurons exhibited ultra-sparse bursting patterns as well, we calculated the maximum number of bursts per trial for each HVC projection neuron. In juveniles, we observed a greater number of bursts per motif per HVC projection neuron compared to adults (maximum number of bursts per motif: juveniles = 0–4 bursts per motif, median = 2, median absolute deviation (MAD) = 1, 10 neurons in 4 juveniles; adults = 0–4 bursts per motif, median = 1, MAD = 0.74, 54 neurons in 10 adults, p = 0.03, Wilcoxon rank sum test, Fig. 1C). Next, we measured the degree of stereotypy in bursting activity across song motifs. We identified bursts as recurring across song motifs (i.e. bursts that occurred reliably across song motifs) if their onset time was within ± 20 ms across motifs. In adults, 85.94% of all recorded bursts were consistently repeated across multiple renditions of the song motif, indicating a high degree of stereotypy. Conversely, in juveniles, only 60% of bursts were recurring (recurring bursts: juveniles = 12/20 bursts, adults = 55/64 bursts, p = 0.02, Fisher Exact Test). These observations are consistent with previous findings [11] and suggest that connectivity within the HVC is less stable and still undergoing plastic changes in late-stage juveniles compared to adults. This could contribute to greater temporal variability in their songs.

In addition to bursting activity, some neurons also exhibited single action potentials during song production, which reduces the sparseness of the neural code and might lead to less reliable behavioral outcomes in terms of song consistency and timing. To test whether single action potentials might contribute to a less temporally stereotyped song, we explored the spiking activity of the HVC neurons, including the total number of spikes during a song motif, spikes within a burst, and the number of single spikes. We observed that the number of spikes per motif tended to be higher in juvenile birds than in adults, although this difference was not statistically significant (spikes per motif: juveniles mean ± standard deviation = 7.1 ± 4.72 spikes/motif, adults mean ± standard deviation = 4.45 ± 4.23 spikes/motif, p = 0.08, two-sample t-test, Fig. 1D). The higher number of spikes per motif could also be attributed to single spikes occurring outside of bursts. To account for the potential impact of single spikes, we separately analyzed their occurrence across song renditions. This analysis revealed that HVC neurons in juvenile zebra finches exhibited a significantly higher number of single action potentials compared to adults (single spikes per motif: juveniles mean ± standard deviation = 1.5 ± 2.56 single spikes/motif, adults mean ± standard deviation = 0.48 ± 1 single spikes/motif, p = 0.03, two-sample t-test, Fig. 1E). The number of spikes recruited for bursting activity per song motif did not differ between juveniles and adults at the single-neuron level (number of spikes within bursts: juveniles mean ± standard deviation = 5.5 ± 3.54, adults mean ± standard deviation = 3.91 ± 3.96, p = 0.23, two-sample t-test, Fig. 1F). These results show that, in late-stage development, HVC projection neurons in juveniles exhibit detectable differences in spiking activity compared to adults. However, this increased spiking activity in juveniles is not reflected in the spiking patterns within the bursts themselves. Instead, it can be attributed to a higher frequency of single spikes occurring outside of bursts. This increased incidence of single spikes might contribute to the greater variability observed in juvenile song production. Next, we explored whether the song variability in juveniles during late-state development could also be attributed to temporal dynamics within bursts in HVC projection neurons.

### Temporal dynamics of HVC neuron bursting activity are slower in juveniles compared to adults

**Fig. 2:**
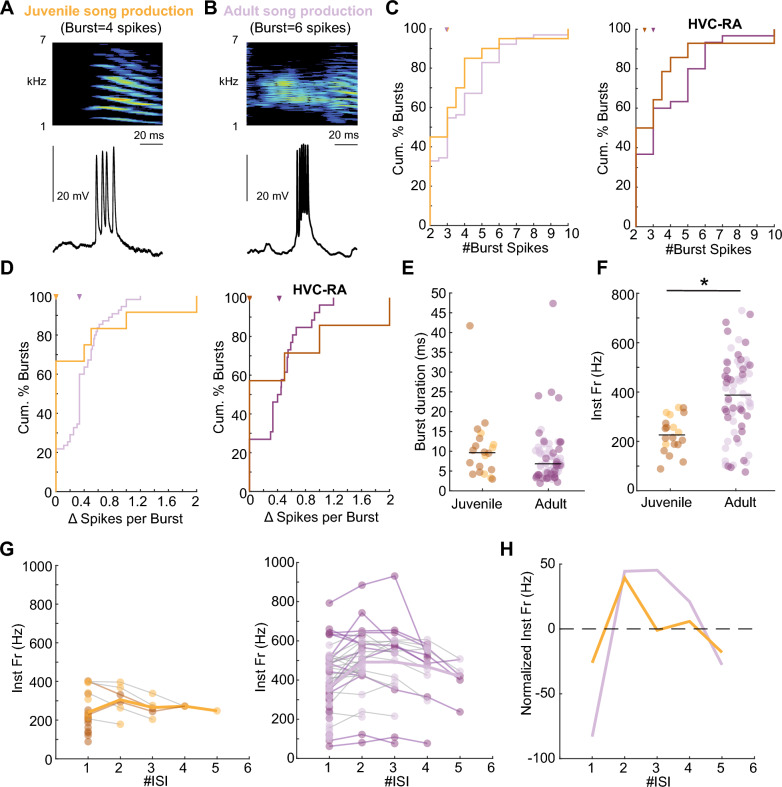
Temporal dynamics within HVC projection neuron bursts. **A** Example of a burst in a juvenile during song production (top: spectrogram with a syllable, below: intracellular recording of a single burst), **B** same as in **A** but for an adult bird. **C** (Left) number of spikes within a burst of all HVC projection neurons in juveniles and adults. (Right) number of spikes within a burst of HVC-RA neurons. Juveniles’ median = 2.5, MAD = 1.37 spikes per burst, Adults median = 3, MAD = 1.65 spikes per burst, Wilcoxon rank sum test, p = 0.34. **D** (Left) distribution of Δ spikes per burst in recurring bursts of all HVC projection neurons in juveniles and adults. (Right) distribution of Δ spikes per burst in recurring bursts of HVC-RA. Juveniles median = 0, MAD = 0.57, Adults’ median = 0.42, MAD = 0.26, Wilcoxon rank sum test, p = 0.64. **E** Burst duration of all recorded bursts in juveniles and adults. Data from HVC-RA neurons are marked in dark shading. **F** Instantaneous firing rate of all bursts in juveniles and adults. HVC-RA projection neurons in both juveniles and adults are marked in dark shading. **G** Progression of instantaneous firing rate in juveniles (left) and adults (right), bold line: mean instantaneous firing rate per number of ISI in juveniles (orange) and adults (purple). Darker lines indicate HVC-RA projection neurons. **H** Progression of instantaneous firing rate from **E** and **F** across ISI

We explored the intrinsic dynamics of individual bursts by analyzing the number of spikes and temporal characteristics of these spikes during song production (Fig. 2A, B). First, we quantified the number of spikes per burst. In both juveniles and adults, we observed a similar median number of spikes per burst (spikes per burst: juveniles median = 3, MAD = 1.32, adults median = 3, MAD = 1.55, p = 0.26, Wilcoxon rank sum test, Fig. 2C). Next, we assessed the variability in the number of spikes in each recurring burst. HVC projection neurons in juveniles had a comparable distribution of Δ number of spikes per burst to that of the neurons in adults (Δ number of spikes per burst: juveniles median = 0, MAD = 0.43 adults median = 0.33, MAD = 0.22, p = 0.09, Wilcoxon rank sum test, Fig. 2D), indicating stereotyped recurring bursts during late-stage development. Next, we investigated whether the temporal structure of bursts in juveniles and adults differed by quantifying their duration. Unlike a previous report [11], we did not observe differences in the duration of bursts between juveniles and adults at a population level, potentially due to our smaller sample size (burst duration: juveniles median = 9.64 ms, MAD = 5.11 ms, adults median = 6.83 ms, MAD = 4.33 ms, Wilcoxon rank sum test, p = 0.15, Fig. 2E). However, when assessing the instantaneous firing rate within bursts, we found that it was lower in juveniles than in adults (firing rate: juveniles median = 226.09 Hz, MAD = 59.35 Hz, adults median = 387.68 Hz, MAD = 135.29 Hz, p < 0.001, Wilcoxon rank sum test, Fig. 2F) indicating that HVC projection neurons are less excitable in juveniles compared to adults. To determine whether the lower instantaneous firing rate produced a distinct temporal pattern of spiking progression within bursts, we next compared the normalized pattern of spiking progression in juveniles and adults (Fig. 2G). After accounting for the overall higher instantaneous firing rate in adults by subtracting the mean instantaneous firing rate trajectory (Fig. 2G) from the mean progression pattern in juveniles and adults, the relative progression pattern in juveniles was indistinguishable from that in adult HVC projection neurons (firing rate: juveniles median = − 1.023 Hz, MAD = 18.07 Hz, adults median = 21.01 Hz, MAD = 44.28 Hz, p = 0.84, Wilcoxon rank sum test, Fig. 2H).

**Fig. 3:**
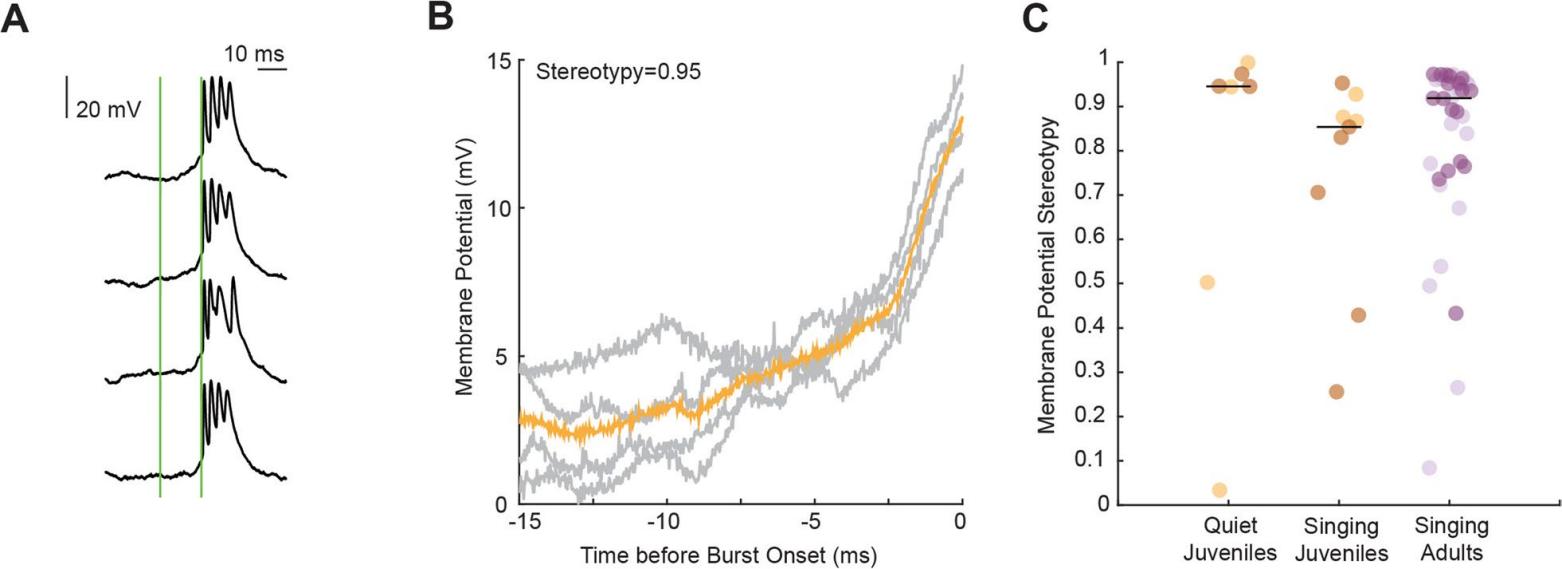
Subthreshold activity preceding bursts. **A** Example recording of an HVC projection neuron in a singing juvenile bird (78 days post hatch), aligned to burst onsets across four song motifs. Green lines indicate the selected interval of analysis 15 ms before burst onset. **B** Subthreshold activity from the four traces (grey) of the example in **A**, 15 ms window before burst onset. In orange—average subthreshold activity of the 15 ms interval. **C** Mean subthreshold stereotypy of all pre-burst intervals per recorded neuron (quiet juveniles: median = 0.94, MAD = 0.28; singing juveniles: median = 0.85, MAD = 0.19; singing adults: median = 0.92, MAD = 0.15), black lines indicate median values of each group. Darker color dots highlight the HVC-RA projectors in both juveniles and adults

To verify whether synaptically connected neurons provide stereotyped excitatory inputs preceding bursts, we analyzed the membrane potential rise during a 15 ms window before all recorded bursts. The excitatory input contributing to the membrane potential rise before bursts was as stereotyped in singing juveniles as in quiet juveniles, where we elicited bursts using current injection (Wilcoxon rank sum test, p = 0.21) or in singing adults (Wilcoxon rank sum test, p = 0.11, Fig. 3). This finding indicates that burst elicitation in singing juveniles occurs in a stereotyped manner comparable to that in adults. The overall stereotyped characteristics of bursts (i.e., number of spikes, burst duration, and stereotyped excitatory input) and the lower instantaneous firing rate suggest precise but slower signal transmission in juvenile HVC projection neurons compared to adults. We hypothesized that these temporal dynamics might also be exhibited in a focal microcircuit within HVC. Therefore, we quantified the synaptic inputs they received. This metric reflects the integrated input a focal neuron receives from its presynaptic network.

### HVC projection neurons receive temporally distinct subthreshold inputs in juveniles compared to adults

**Fig. 4:**
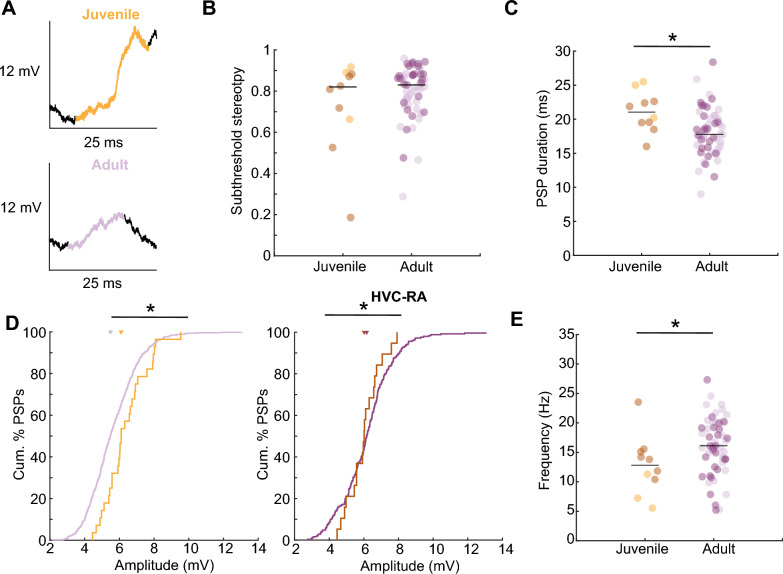
Temporal features of the subthreshold activity. **A** Example of excitatory post-synaptic event detection in juveniles (orange) and adults (purple). **B** Subthreshold activity exhibited high stereotypy in juveniles and adults. Data from HVC-RA neurons marked in dark. **C** Mean PSP duration per HVC projection neuron. **D** (Left) distribution of amplitude of all excitatory PSP events recorded in all HVC projection neurons. (Right) distribution of amplitude of all excitatory PSP events recorded in HVC-RA neurons. Juveniles’ median = 6.02 mV, MAD = 0.71 mV, Adults’ median = 6.16 mV, MAD = 1.16 mV Wilcoxon rank sum test p = 0.004. **E** Mean frequency of all PSP events per HVC projection neuron

To quantify the temporal dynamics of inputs that HVC projection neurons are receiving, we assessed the stereotypy of the membrane potential stereotypy by correlating subthreshold activity across motif renditions for each recorded neuron (Fig. 4A). In juveniles, the subthreshold activity was as stereotyped as in adults (subthreshold precision: juveniles median = 0.82, MAD = 1.64, adults median = 0.83, MAD = 0.09, p = 0.59, Wilcoxon rank sum test, Fig. 4B), suggesting a stable neural representation of song production. Since we previously reported temporal differences in bursting activity, we hypothesized that these differences could also be reflected in the summation of excitatory and inhibitory postsynaptic potentials (PSPs) received by a focal neuron. To address these temporal dynamics, we quantified the duration of the PSPs and observed that PSP events in juveniles were of longer duration than in adults (PSP duration: juveniles median = 21.04 ms, MAD = 2.36, adults median = 17.79 ms, MAD = 2.68, p = 0.01, Wilcoxon rank sum test, Fig. 4C), which is in line with our previously observed slower temporal dynamics within bursts in juveniles. Further we quantified the membrane potential amplitude and found that PSPs in juveniles had a higher amplitude than in adults (PSP amplitude: juveniles median = 6.10 mV, MAD = 0.96, adults median = 5.49 mV, MAD = 1.12, p < 0.01, Wilcoxon rank sum test, Fig. 4D), which could potentially be attributed to a less negative resting membrane potential in juveniles [29]. In juveniles, a less negative resting membrane potential is associated with altered neuronal excitability and could likely manifest as changes in the frequency or amplitude of postsynaptic potential events. The frequency of the PSP elements was lower in juveniles than in adults (PSP element frequency: juveniles median = 12.82 Hz, MAD = 3.59, adults median = 16.12 Hz, MAD = 3.85, p = 0.045, Wilcoxon rank sum test, Fig. 4E), suggesting a slower-paced, more sparse input from synaptically connected neurons. The slower and more sparse membrane potential dynamics during singing in juveniles might explain the temporal differences observed during song performance [4, 23].

## Discussion

In this study, we explored the transition from the variable plastic song of juvenile zebra finches to the crystallized song of adults to investigate the neural mechanisms underlying this behavioral shift. We focused specifically on HVC, a premotor nucleus critical for song learning and production, to understand the neural dynamics that facilitate this transformation. The song crystallization phase in late-stage juveniles is characterized by a higher number of bursts in HVC projection neurons during singing, which were more variable in their occurrence than bursts of HVC projection neurons in adult birds (Fig. 1). This neural pattern may underlie the more variable and exploratory vocalizations observed in juveniles during song learning. Despite receiving stereotyped excitatory input, bursts in juveniles exhibited a lower instantaneous firing rate than those in adults (Fig. 2). This finding suggests that developmental changes in intrinsic neuronal properties may play a role in how the temporal information is encoded within HVC. Furthermore, the stereotyped postsynaptic events occurring at a lower frequency in juveniles (Fig. 3) may indicate slower dynamics in their excitatory drive.

In adult birds, HVC-RA projection neurons produce sparse bursts during singing [14]. These bursts are triggered by presynaptic inputs with varying latencies, forming neural sequences during song production [19]. In juvenile birds, HVC connectivity develops over time, with bursting events gradually becoming more timepoint-specific during singing [11]. In this study, we report that bursting activity in late-stage juveniles occurred more frequently and with less stereotypy across motifs than in adults. Song development in zebra finches is accompanied by a progressive increase in inhibition within the HVC [30]. In adult birds, precise inhibitory neuron activity is crucial for shaping the sparse bursts of excitatory neurons within HVC, which likely contributes to the stereotyped nature of adult song [31]. It is plausible that the excitatory-inhibitory balance within the HVC of the juvenile birds we recorded was not yet fully matured, potentially contributing to the observed differences in bursting activity compared to adults.

In terms of subthreshold activity, neural dynamics were stable during song production in both juveniles and adults, consistent with a previous study reporting highly stereotyped female-directed songs in juveniles, which were more variable during song practice [32]. Additionally, the higher amplitude post-synaptic potentials in juveniles may indicate lower inhibitory current within HVC during song performance. Alternatively, neural dynamics may exhibit distinct temporal signatures due to differences in the membrane properties of the HVC projection neurons in juveniles and adults. During development, the membrane properties of HVC projection neurons show a higher resting membrane potential and increased spiking amplitude [29], which could lead to a greater general excitability of the HVC projection neurons. Supporting this hypothesis, we report an increased amplitude of excitatory postsynaptic events in juveniles. These excitatory post-synaptic events occurred at a lower frequency during singing in juveniles compared to adults. The slower temporal dynamics could be attributed to incomplete myelination of axonal connections and changing membrane properties during development [29], ultimately impacting signal propagation within HVC during the juvenile stage. Consistent with this hypothesis, studies in adults demonstrate relatively slow conduction velocities in HVC projection neurons [19]. Additionally, the intrinsic properties of a subset of HVC projection neurons show greater variability during song learning than in adulthood [33], likely contributing to increased variability in HVC neural dynamics during song production. Investigating signal propagation within the HVC during development could yield further insights into its role in HVC network dynamics during song learning.

We identified distinct temporal dynamics within the HVC during the song crystallization phase, where the temporal and spectral features of the song are fine-tuned. Despite the differences in the temporal dynamics of HVC projection neurons between the song crystallization phase and adulthood, the overall stereotypy of neural patterns during song production remained stable. The observed differences in spiking and subthreshold activity between juvenile and adult HVC projection neurons underscore the dynamic nature of neural circuits during song learning. These findings suggest that HVC undergoes significant plasticity during development to establish the stable, stereotyped patterns associated with mature song production.

## Materials and methods

### Animal housing

All procedures were approved by the Regierungspräsidium Oberbayern (VET 02-21-201), Landesamt für Gesundheit und Soziales (LAGeSo Berlin) (G 0225/16) at the Freie Universität Berlin. Juvenile zebra finches (n = 4, older than 73 dph) were housed in an aviary or a breeding cage with their genetic parents up to 60 dph, at which time, they were moved to an adjacent aviary where they had visual and auditory contact with their parents. Adult male birds (n = 10, older than 100 dph) were acquired from Magnolia Bird Farm. All maintenance and experimental procedures of adult birds were performed according to the guidelines established by the Institutional Animal Care and Use Committee at New York University Langone Medical Center. The adult data set was previously recorded at New York University School of Medicine in Michael Long’s laboratory and was published in Vallentin and Long [28].

### Surgery

Male zebra finches were anesthetized with isoflurane (concentration: 1–3% isoflurane, 97–99% oxygen). Subsequently, they were carefully secured to a stereotactic device using ear bars, ensuring their heads were positioned at a 65° angle in relation to the horizontal plane. This angle was determined by measuring the beak angle. An incision was made to expose the skull, and a square shaped area of trabecular bone was removed above HVC, RA and cerebellum using a dental drill (carbide bur, FG ¼, Johnson-Promident). Nucleus RA was targeted according to coordinates (0 point at the bifurcation of the midsagittal sinus, RA coordinates: posterior 1.85 mm, lateral 2.25 mm, ventral 1.8 mm). A carbon fiber electrode (Kation Scientific, LLC) was used to identify RA based on the firing pattern [18, 34]. Next, we targeted HVC using coordinates (0 point at the bifurcation of the midsagittal sinus, HVC coordinates: anterior 0.2 mm, lateral 2.3 mm, ventral 0.2 mm) and confirmed the location with antidromic stimulation from RA [14]. A previously assembled microdrive for intracellular recordings [27] was then implanted at an 25° angle (relative to the right angle of the horizontal plane) above HVC for intracellular recordings. Birds were let to recover for 1–3 days before the intracellular recordings.

### Intracellular recordings in freely behaving animals

To record intracellularly we used sharp intracellular electrodes (borosilicate glass with filament, 0.1 mm diameter), that were previously pulled using a micropipette puller (Model P-97, Sutter Instrument). Before use, each electrode was backfilled with potassium acetate (concentration: 3 M). Using a silicone elastomer, we built a well around the craniotomy in HVC and filled it with phosphate buffered saline (PBS). Next, we removed dura using a dura pick. While birds were in a head-fixed setting, we lowered down the glass electrode in HVC until we could identify surrounding cells using oscilloscope and an audio monitor. We then transferred the bird into the cage of the recording setup and gradually lowered the electrode within HVC, accompanied by a brief buzzing pulse (10–20 ms), until a successful penetration of a neuron. Next, we presented a female bird to the male bird to motivate singing behavior. For this study, we only selected neurons that had at least 30 mV action potentials, the recording lasted at least 3 min, and the membrane potential was below − 50 mV. To maintain consistency in recording quality, we applied identical criteria to the data collected from adult birds. Neurons were categorized as HVC-RA neurons if they exhibited either of the following characteristics: an antidromic spike was triggered upon electrical stimulation of RA [14], or the neuron depolarized during song production [27]. Conversely, neurons that hyperpolarized during singing were classified as HVC-Area X neurons [27]. Based on previously reported firing statistics [35] we did not record from HVC interneurons.

### Spike and burst detection

Spikes in each recorded trace were detected as events exceeding the 15 mV threshold from the averaged trace. We defined bursts as single events where the firing rate of adjacent spikes exceeded 100 Hz and the spikes occurred on the same membrane depolarization event. To compare recurring bursts, we defined a burst as recurring, if the onset time of the burst (first spike) in next trial occurred ± 20 ms from the onset of burst in the previous trial. The difference in the number of spikes compared to the most common spike count observed in the reference condition per burst was calculated for each identified, recurring burst. First, the mode of spikes per burst was calculated for each recurring burst. Then, the number of spikes of each burst of the same identity was subtracted from mode and an absolute average difference of number of spikes was calculated per identified burst. For the 15 ms depolarization events occurring prior bursts, we analyzed data from previously recorded quiet juvenile birds (n = 7 neurons in 5 birds), singing juvenile birds (n = 10 neurons in 4 birds) and adult birds (n = 37 neurons in 10 birds).

### Instantaneous firing rate

Instantaneous firing rate was calculated as the mean firing rate per recurring burst and was defined as number of inter spike interval (ISI) divided by the sum of ISIs per burst. To make a fair comparison of the temporal progression of spiking within bursts, we considered only those bursts with no more than 6 spikes per burst for this analysis.

### Excitatory post-synaptic potential detection

For detection of the PSPs, we first detected local peaks and events of membrane depolarization exceeding 2 mV amplitude, then measured their amplitude and calculated the frequency by dividing the number of events with the duration of the song motif. For the analysis of PSP duration, we included only those PSPs that were shorter than 35 ms to avoid false PSP duration detection.

### Subthreshold stereotypy

Subthreshold stereotypy was defined as a cross correlation at 0 lag of all trials within a cell. First, we cut the spikes of our recorded traces (spike detection as described above). We then demeaned all subthreshold traces for a fair comparison. Each trace was then tested for a correlation with every other trace from the same HVC projection neuron.

### Euthanasia protocol

Following the experiment, birds were euthanized via the administration of an overdose of isoflurane.

## Data Availability

All custom scripts including example data are available here: https://github.com/vallentinlab/HVC-dynamics-in-zebra-finches. Due to space limitations of the public repository, the complete neural recordings will be made available upon request. Please contact the corresponding author.

## References

[1] Oller DK. Chapter 6—The emergence of the sounds of speech in infancy. In: Yeni-komshian GH, Kavanagh JF, Ferguson CA, editors. Child phonology. New York: Academic Press; 1980. p. 93–112. 10.1016/B978-0-12-770601-6.50011-5.

[2] Lightfoot D. Language acquisition and language change. WIREs Cognit Sci. 2010;1:677–84. 10.1002/wcs.39 26271652

[3] Doupe AJ, Kuhl PK. Birdsong and human speech: common themes and mechanisms. 1998. www.annualreviews.org. 10.1146/annurev.neuro.22.1.567 10202549

[4] Tchernichovski O, Mitra PP, Lints T, Nottebohm F. Dynamics of the vocal imitation process: how a zebra finch learns its song. Science. 2001;291:2564–9. 11283361 10.1126/science.1058522

[5] Tchernichovski O, Nottebohm F, Ho CE, Pesaran B, Mitra PP. A procedure for an automated measurement of song similarity. Anim Behav. 2000;59:1167–76. 10877896 10.1006/anbe.1999.1416

[6] Derégnaucourt S, Poirier C, der Kant AV, der Linden AV, Gahr M. Comparisons of different methods to train a young zebra finch (*Taeniopygia guttata*) to learn a song. J Physiol Paris. 2013;107:210–8. 22982543 10.1016/j.jphysparis.2012.08.003

[7] Zann RA. The zebra finch—a synthesis of field and laboratory studies. Oxford: Oxford University Press; 1996. p. 197–247.

[8] Ölveczky BP, Otchy TM, Goldberg JH, Aronov D, Fee MS. Changes in the neural control of a complex motor sequence during learning. J Neurophysiol. 2011;106:386–97. 21543758 10.1152/jn.00018.2011 PMC3129720

[9] Shank SS, Margoliash D. Sleep and sensorimotor integration during early vocal learning in a songbird. Nature. 2009;458:73–7. 19079238 10.1038/nature07615 PMC2651989

[10] Aronov D, Andalman AS, Fee MS. A specialized forebrain circuit for vocal babbling in the juvenile songbird. Science. 2008;320:630–4. 18451295 10.1126/science.1155140

[11] Okubo TS, Mackevicius EL, Payne HL, Lynch GF, Fee MS. Growth and splitting of neural sequences in songbird vocal development. Nature. 2015;528:352–7. 26618871 10.1038/nature15741 PMC4957523

[12] Mackevicius EL, Gu S, Denisenko NI, Fee MS. Self-organization of songbird neural sequences 1 during social isolation 2. Elife. 2023. 10.1101/2022.02.18.480996. 37252761 10.7554/eLife.77262 PMC10229124

[13] Aronov D, Veit L, Goldberg JH, Fee MS. Two distinct modes of forebrain circuit dynamics underlie temporal patterning in the vocalizations of young songbirds. J Neurosci. 2011;31:16353–68. 22072687 10.1523/JNEUROSCI.3009-11.2011 PMC3241969

[14] Hahnloser RHR, Kozhevnikov AA, Fee MS. An ultra-sparse code underliesthe generation of neural sequences in a songbird. Nature. 2002;419:65–70. 12214232 10.1038/nature00974

[15] Picardo MA, et al. Population-level representation of a temporal sequence underlying song production in the zebra finch. Neuron. 2016;90:866–76. 27196976 10.1016/j.neuron.2016.02.016 PMC4941616

[16] Lynch GF, Okubo TS, Hanuschkin A, Hahnloser RHR, Fee MS. Rhythmic continuous-time coding in the songbird analog of vocal motor cortex. Neuron. 2016;90:877–92. 27196977 10.1016/j.neuron.2016.04.021

[17] Leonardo A, Fee MS. Ensemble coding of vocal control in birdsong. J Neurosci. 2005;25:652–61. 15659602 10.1523/JNEUROSCI.3036-04.2005 PMC6725314

[18] Yu AC, Margoliash D. Temporal hierarchical control of singing in birds. New Ser. 1996;273:1871–5. 10.1126/science.273.5283.1871 8791594

[19] Egger R, et al. Local axonal conduction shapes the spatiotemporal properties of neural sequences. Cell. 2020;183:537-548.e12. 33064989 10.1016/j.cell.2020.09.019 PMC7577554

[20] Wild JM. Functional neuroanatomy of the sensorimotor control of singing. Ann N Y Acad Sci. 2004;1016:438–62. 15313789 10.1196/annals.1298.016

[21] Mehaffey WH, Doupe AJ. Naturalistic stimulation drives opposing heterosynaptic plasticity at two inputs to songbird cortex. Nat Neurosci. 2015;18:1272–80. 26237364 10.1038/nn.4078 PMC5726397

[22] Ölveczky BP, Andalman AS, Fee MS. Vocal experimentation in the juvenile songbird requires a basal ganglia circuit. PLoS Biol. 2005;3:0902–9. 10.1371/journal.pbio.0030153 PMC1069649 15826219

[23] Glaze CM, Troyer TW. Development of temporal structure in zebra finch song. J Neurophysiol. 2013;109:1025–35. 23175805 10.1152/jn.00578.2012 PMC3569136

[24] Long MA, Fee MS. Using temperature to analyse temporal dynamics in the songbird motor pathway. Nature. 2008;456:189–94. 19005546 10.1038/nature07448 PMC2723166

[25] Scharff C, Nottebohm F. A comparative study of the behavioral deficits following lesions of various parts of the zebra finch song system: implications for vocal learning. J Neurosci. 1991;11:2896. 1880555 10.1523/JNEUROSCI.11-09-02896.1991 PMC6575264

[26] Chen JR, Stepanek L, Doupe AJ. Differential contributions of basal ganglia and thalamus to song initiation, tempo, and structure. J Neurophysiol. 2014;111:248–57. 24174647 10.1152/jn.00584.2012 PMC3921389

[27] Long MA, Jin DZ, Fee MS. Support for a synaptic chain model of neuronal sequence generation. Nature. 2010;468:394–9. 20972420 10.1038/nature09514 PMC2998755

[28] Vallentin D, Long MA. Motor origin of precise synaptic inputs onto forebrain neurons driving a skilled behavior. J Neurosci. 2015;35:299–307. 25568122 10.1523/JNEUROSCI.3698-14.2015 PMC4287148

[29] Ross MT, Flores D, Bertram R, Johnson F, Hyson RL. Neuronal intrinsic physiology changes during development of a learned behavior. eNeuro. 2017. 10.1523/ENEURO.0297-17.2017. 29062887 10.1523/ENEURO.0297-17.2017 PMC5649544

[30] Vallentin D, Kosche G, Lipkind D, Long MA. Neural circuits: inhibition protects acquired song segments during vocal learning in zebra finches. Science. 2016;351:267–71. 26816377 10.1126/science.aad3023 PMC4860291

[31] Kosche G, Vallentin D, Long MA. Interplay of inhibition and excitation shapes a premotor neural sequence. J Neurosci. 2015;35:1217–27. 25609636 10.1523/JNEUROSCI.4346-14.2015 PMC4300325

[32] Kojima S, Doupe AJ. Social performance reveals unexpected vocal competency in young songbirds. Proc Natl Acad Sci. 2011;108:1687–92. 21220335 10.1073/pnas.1010502108 PMC3029722

[33] Daou A, Margoliash D. Intrinsic neuronal properties represent song and error in zebra finch vocal learning. Nat Commun. 2020;11:952. 32075972 10.1038/s41467-020-14738-7 PMC7031510

[34] Spiro JE, Dalva MB, Mooney R. Long-range inhibition within the zebra finch song nucleus RA can coordinate the firing of multiple projection neurons. J Neurophysiol. 1999;81:3007–20. 10368416 10.1152/jn.1999.81.6.3007

[35] Kozhevnikov AA, Fee MS. Singing-related activity of identified HVC neurons in the zebra finch. J Neurophysiol. 2007;97:4271–83. 17182906 10.1152/jn.00952.2006

